# A novel prognostic signature related to programmed cell death in osteosarcoma

**DOI:** 10.3389/fimmu.2024.1427661

**Published:** 2024-07-01

**Authors:** Yu-Chen Jiang, Qi-Tong Xu, Hong-Bin Wang, Si-Yuan Ren, Yao Zhang

**Affiliations:** ^1^ Affiliated Zhongshan Hospital Of Dalian University, Dalian, China; ^2^ Department of Gastrointestinal Surgery, Shanghai East Hospital, School of Medicine, Tongji University, Shanghai, China

**Keywords:** osteosarcoma, tumor immunology, programmed cell death, machine learning algorithm, prognostic marker

## Abstract

**Background:**

Osteosarcoma primarily affects children and adolescents, with current clinical treatments often resulting in poor prognosis. There has been growing evidence linking programmed cell death (PCD) to the occurrence and progression of tumors. This study aims to enhance the accuracy of OS prognosis assessment by identifying PCD-related prognostic risk genes, constructing a PCD-based OS prognostic risk model, and characterizing the function of genes within this model.

**Method:**

We retrieved osteosarcoma patient samples from TARGET and GEO databases, and manually curated literature to summarize 15 forms of programmed cell death. We collated 1621 PCD genes from literature sources as well as databases such as KEGG and GSEA. To construct our model, we integrated ten machine learning methods including Enet, Ridge, RSF, CoxBoost, plsRcox, survivalSVM, Lasso, SuperPC, StepCox, and GBM. The optimal model was chosen based on the average C-index, and named Osteosarcoma Programmed Cell Death Score (OS-PCDS). To validate the predictive performance of our model across different datasets, we employed three independent GEO validation sets. Moreover, we assessed mRNA and protein expression levels of the genes included in our model, and investigated their impact on proliferation, migration, and apoptosis of osteosarcoma cells by gene knockdown experiments.

**Result:**

In our extensive analysis, we identified 30 prognostic risk genes associated with programmed cell death (PCD) in osteosarcoma (OS). To assess the predictive power of these genes, we computed the C-index for various combinations. The model that employed the random survival forest (RSF) algorithm demonstrated superior predictive performance, significantly outperforming traditional approaches. This optimal model included five key genes: MTM1, MLH1, CLTCL1, EDIL3, and SQLE. To validate the relevance of these genes, we analyzed their mRNA and protein expression levels, revealing significant disparities between osteosarcoma cells and normal tissue cells. Specifically, the expression levels of these genes were markedly altered in OS cells, suggesting their critical role in tumor progression. Further functional validation was performed through gene knockdown experiments in U2OS cells. Knockdown of three of these genes—CLTCL1, EDIL3, and SQLE—resulted in substantial changes in proliferation rate, migration capacity, and apoptosis rate of osteosarcoma cells. These findings underscore the pivotal roles of these genes in the pathophysiology of osteosarcoma and highlight their potential as therapeutic targets.

**Conclusion:**

The five genes constituting the OS-PCDS model—CLTCL1, MTM1, MLH1, EDIL3, and SQLE—were found to significantly impact the proliferation, migration, and apoptosis of osteosarcoma cells, highlighting their potential as key prognostic markers and therapeutic targets. OS-PCDS enables accurate evaluation of the prognosis in patients with osteosarcoma.

## 1 Introduction

Osteosarcoma is an exceptionally aggressive cancer that develops in bone tissue, predominantly affecting children and teenagers. This malignancy constitutes nearly half of all bone cancers in this age group. It commonly arises in the metaphyseal regions of long bones, such as the distal femur, proximal tibia, and proximal humerus (1). Characteristically, osteosarcoma is marked by uncontrolled proliferation of bone and cartilage tissue, leading to significant morbidity (2). Recent statistical data indicate that the average 5-year survival rate for osteosarcoma patients is around 70%. However, this rate significantly declines to below 30% for those with distant metastases. The pathogenesis of osteosarcoma involves complex genetic alterations that drive the initiation and progression of the tumor (3).

Osteosarcoma is characterized by aberrant gene expression, which exerts significant influence on the initiation and progression of tumor cells (4, 5). Key molecular pathways implicated in osteosarcoma include the STING/IRF3/IFN-β, PI3K/AKT, and mTOR signaling pathways (6–8). Considering the complexity of osteosarcoma pathogenesis and the heterogeneity of its molecular landscape, identifying critical genes affecting prognosis and constructing a simple yet effective prognostic model is paramount.

With the advancement of cancer biology research, the interaction between programmed cell death (PCD) and malignant tumors has received widespread attention, being considered a crucial component in the occurrence of malignant tumors (9, 10). As research progresses, an increasing number of researchers are focusing on the correlation between PCD and osteosarcoma occurrence (11). Apoptosis, the predominant form of cell death, is essential for the proper functioning of organisms. Abnormal activation of apoptosis pathways may lead to sustained proliferation and abnormal survival of tumor cells, which could promote tumor occurrence and growth (12). Ferroptosis, another form of cell death induced by oxidative stress, has attracted significant interest in the realm of cancer research. Due to the weakened antioxidant capacity of tumor cells, they are more susceptible to iron-dependent cell death (13). Cuproptosis has also attracted researchers’ interest, as this form of death may be related to the death and survival of tumor cells (14). Autophagy is an intracellular degradation process crucial for maintaining cellular homeostasis, as it breaks down damaged proteins and organelles (15). PARP-1 is an important factor associated with DNA damage repair and the effectiveness of cancer drugs (16). Pyroptosis is an inflammatory type of cell death that generally happens after the involved cells release cytokines like interleukins and other signaling molecules (17). By aiding the decline of inflammatory cells to control tumor progression and, to a certain extent, modifying the response to treatment (18). Netotic cell death, a distinct form of cellular demise, is triggered by the formation and accumulation of net-like fibrous structures (19). The possibility of this kind of regulation lies in an apoptotic function of the tumor microenvironment. They are interconnected with the consequences of oxidative harm (20). Anoikis is a type of cellular demise initiated by the separation of cells from the extracellular matrix, Dysregulation of extracellular matrix interaction may impact tumor cell survival and metastasis (21). Immunogenic cell death is a type of cellular demise that can provoke an immune response, consequently impacting the survival of tumor cells (22). Disulfidptosis is a type of cell death linked to the formation and disruption of intracellular disulfide bonds. This process may also contribute to tumor initiation and progression (23). In summary, a deeper comprehension of the different forms of programmed cell death and their roles in the development of malignant tumors will yield new insights and targets for cancer prevention and therapy. Further research is essential to explore how these apoptotic pathways function in cancer treatment, aiming to enhance the survival rates and quality of life for cancer patients.

The creation of prognostic models for osteosarcoma has been greatly enhanced by the advancement of machine learning methods. When it comes to understanding the complexity of biological information, the application of different algorithms provides special advantages within the field of machine learning. A selection of 10 algorithms renowned for their intrinsic variable selection properties has been made, encompassing Enet, Ridge, CoxBoost, survivalSVM, Lasso, SuperPC, plsRcox, StepCox, RSF, and GBM. Lasso is a regression analysis method used for feature selection and model sparsity, aiding in identifying key predictor variables (24). Ridge regression constrains the size of model coefficients by adding an L2 regularization term, reducing overfitting, particularly useful for handling collinear data (25). Elastic Net combines the strengths of Lasso and Ridge, balancing model sparsity and complexity, suitable for high-dimensional and collinear data (26). CoxBoost is a Cox proportional hazards model based on gradient boosting trees, used for modeling and predicting survival data (27). Utilizing support vector machines to capture intricate relationships between variables and survival time, Survival Support Vector Machine is a technique for managing survival data (28). With the advancement of machine learning integration, the establishment of prognosis models for different diseases has significantly improved. The application of various algorithms in the field of machine learning provides distinct advantages. Ten algorithms known for their inherent variable selection capabilities include Enet, Ridge, CoxBoost, survivalSVM, Lasso, SuperPC, plsRcox, StepCox, RSF, and GBM. By integrating these methods, the intricate relationship between programmed cell death (PCD) and osteosarcoma (OS) prognosis can be more thoroughly understood. The complementary nature of these algorithms greatly enhances the model’s generalizability and predictive accuracy.

This research employed the integration of several machine learning algorithms to create a prognostic model for osteosarcoma associated with programmed cell death (PCD). The underlying assumption was that combining various algorithms would better capture the complex gene interactions and enhance prediction accuracy. The goal was to improve the accuracy of prognostic predictions for osteosarcoma patients and to identify potential targets for personalized treatment strategies.

## 2 Methods

### 2.1 Data download and standardization

We obtained a comprehensive dataset from the Therapeutically Applicable Research to Generate Effective Treatments (TARGET) database. This dataset contained detailed information on 202 osteosarcoma patients, encompassing expression data, clinical details, and chromosomal and gene mutation information (https://ocg.cancer.gov/programs/target). To ensure the robustness of our study, we applied stringent criteria to screen for samples with complete data, resulting in a selection of 88 osteosarcoma patient samples. Furthermore, we enriched our analysis by incorporating three additional datasets from the Gene Expression Omnibus (GEO) database (https://www.ncbi.nlm.nih.gov/geo/): GSE16102, GSE21257, and GSE39058. These datasets collectively contributed an additional 128 osteosarcoma patient samples. Each dataset was carefully chosen based on its relevance and the completeness of the accompanying clinical annotations.

At the outset of osteosarcoma data analysis, we utilized the IOBR package’s data transformation function to convert the raw count matrix from the TARGET dataset into Transcripts Per Million (TPM) format. This conversion was crucial for normalizing the expression levels across samples. Next, we utilized the NormalizeBetweenArrays function from the limma package to standardize the data, which helped to reduce batch effects and ensure comparability across various datasets. For the GEO datasets, we reversed the log2-transformed count values to restore the original count values before converting them to TPM format. Batch effects were addressed using the movebatcheffect function from the sva package. These stringent normalization steps were essential to maintain the integrity and reproducibility of our results.

Through a thorough literature review, we identified 15 distinct types of programmed cell death, including disulfidptosis, entotic cell death, netotic cell death, pyroptosis, ferroptosis, anoikis, autophagy, necroptosis, PARP-1-dependent cell death, alkaliptosis, oxeiptosis, immunogenic cell death, and lysosome-dependent cell death. The genes associated with these forms of cell death were sourced from the Gene Set Enrichment Analysis (GSEA) database (http://www.gsea-msigdb.org/gsea/index.jsp) and the Kyoto Encyclopedia of Genes and Genomes (KEGG) database (https://www.genome.jp/kegg/). These genes were then combined using the intersection function, resulting in a comprehensive set of 1621 programmed cell death genes.

### 2.2 Selection of differentially expressed genes and identification of prognosis-associated genes

Utilizing the limma software for differential expression analysis, we pinpointed genes significantly linked to programmed cell death (PCD) in 88 osteosarcoma samples and their corresponding adjacent normal tissues from the training set. The differential analysis criteria were established at FDR < 0.05 and |log2Foldchange| > 1. Osteosarcoma samples and their adjacent normal tissues were differentiated using TARGET sample IDs. To visualize the differentially expressed genes, we generated a heatmap by randomly selecting a subset of these genes with the pheatmap package. Additionally, we created a volcano plot using the ggplot2 tool to display the fold changes, p-values, and expression levels of these genes.

In our thorough UniCox regression analysis, conducted with the survival package in R, we identified 30 genes significantly correlated with patient survival, applying a stringent p-value threshold of < 0.05. Out of these, 24 genes were found to have protective effects, while 6 were associated with a higher risk prognosis. These findings were illustrated through detailed forest plots, providing clear depictions of hazard ratios and confidence intervals. To further elucidate the intricate relationships between genes associated with prognosis, we constructed chord plots using the igraph, psych, and reshape2 packages. These plots illustrated complex interactions and co-expression patterns among the identified genes. Additionally, lollipop plots and the Circos package were employed to identify and present prognostic genes with copy number variations (CNVs) greater than 4% in the training set of osteosarcoma samples, offering deeper insights into the genomic alterations and their potential impact on prognosis.

### 2.3 Establishment of osteosarcoma subtypes and mechanism analysis

We utilized the ConsensusClusterPlus package in R to perform unsupervised clustering on the test set of osteosarcoma samples. The maxK parameter was set to 9, generating subtypes ranging from 2 to 9. Through an in-depth examination of the clustering heatmap and the consensus variation curve, we determined the optimal value of K to be 2. Consequently, the training set samples were categorized into two distinct subtypes, labeled as A and B. The consensus clustering results were subsequently validated using t-distributed stochastic neighbor embedding (tSNE) and Uniform Manifold Approximation and Projection (UMAP) techniques. Kaplan-Meier (KM) survival curves for osteosarcoma patients were created using the survival package in R to depict the prognostic differences between these two subtypes.

To explore the underlying mechanisms behind the survival rate disparities between the subtypes, we conducted a differential expression analysis of prognostically relevant genes using the limma package. We utilized single-sample gene set enrichment analysis (ssGSEA) to determine variations in immune cell abundance between the subtypes. To explore differential pathway enrichment between the subtypes, gene set variation analysis (GSVA) was performed, and the top twenty most differentially enriched pathways were visualized using the pheatmap package. Reference gene sets from the KEGG and GSEA databases were employed, and the Gene Set Enrichment Analysis (GSEA) method was applied to identify pathways significantly enriched in each subtype. Furthermore, we illustrated the expression levels of the 30 prognosis-associated genes in subtypes A and B, along with clinical information for each sample.

### 2.4 Development of integrated machine learning models

After a preliminary screening, we identified ten machine learning algorithms known for their excellent variable selection properties. We integrated these into a robust ensemble. The chosen algorithms included Enet, Ridge, CoxBoost, survivalSVM, Lasso, SuperPC, plsRcox, StepCox, RSF, and GBM. Leveraging the corresponding R packages, we established fundamental computational protocols for each model. The selection of these algorithms was based on their strengths in handling high-dimensional data, regularization, and boosting, which are critical for robust prognostic modeling.

During model training, we optimized key parameters for each algorithm. For instance, the Lasso and Ridge regressions included tuning the regularization parameter lambda, which controls the strength of the penalty imposed on the coefficients. The Elastic Net model balanced the l1_ratio parameter to combine the penalties of Lasso and Ridge. For the survivalSVM, we adjusted the cost parameter to manage the trade-off between margin maximization and classification error. In the case of the CoxBoost algorithm, the step size and the number of boosting steps were finely tuned to prevent overfitting while ensuring adequate model complexity.

Subsequently, we implemented a strategy where one algorithm was responsible for variable selection, while another algorithm was tasked with model construction. Pairing these ten algorithms in all possible combinations resulted in 101 combinations. Each combination was rigorously cross-validated, with hyperparameters fine-tuned based on performance metrics like the concordance index (C-index) and mean squared error.

The concordance index (C-index) was computed for each model, considering both survival time and status. To ensure robust performance metrics, k-fold cross-validation was employed for model evaluation. Ultimately, the Osteosarcoma Programmed Cell Death Score (OS-PCDS) model was developed using the Random Survival Forest (RSF) algorithm, which exhibited the lowest relative variability and the highest average C-index across validation sets. The exceptional performance of this model is attributed to RSF’s ability to handle complex variable interactions and its robustness against overfitting through ensemble learning.

By combining the glmnet R utility with the CalRiskScore function, the parameter type was configured to linear predictor. Subsequently, risk scores were computed for each sample in both the training and validation sets. The median OS-PCDS value from the training set was used as the threshold to classify all osteosarcoma (OS) samples in the dataset into high-risk and low-risk groups.

During the variable selection process, the RSF algorithm identified five genes associated with prognosis, which became the foundation of the prognostic model. Box plots were used to illustrate the expression levels of these five genes in osteosarcoma samples from the training set and adjacent normal tissues. Independent survival analyses were subsequently conducted for the high-expression and low-expression groups of these model genes.

### 2.5 Verification of model prediction accuracy and exploration of mechanism

We manually curated 61 transcriptome-based prognostic models for osteosarcoma from publicly available sources, the list of models is provided in **Supplementary Table S1**. In this study, variables were excluded from published models if the proportion of missing model genes in the training set expression matrix exceeded 20%. Consequently, a total of 38 models were selected for comparison. Comparative analyses with previously published osteosarcoma prognostic models involved generating forest plots to represent the C-index for both training and validation sets. Kaplan-Meier (KM) curves, illustrating overall survival across all training and validation sets, were constructed using the survival and survminer R packages. The comparison of survival curves between low-risk and high-risk groups in the training and validation sets was performed using the survdiff function, yielding statistically significant p-values (<0.05). To improve the reliability of prognostic predictions for osteosarcoma patients, ROC (Receiver Operating Characteristic) curves were generated at 1, 2, 3, 4, and 5 years for both the training and validation sets using the timeROC package. Subsequently, the area under the curve (AUC) values were calculated for each ROC curve. Additionally, to validate the prognostic significance of OS-PCDS and explore its potential as a supplementary tool to current clinical data, ROC values were calculated for all clinically relevant information to forecast outcomes at 1-5 years. Following this, the predictive effectiveness of PCDS was evaluated using multiCox regression, which incorporated potential confounding factors from additional clinical data, displayed in forest plot form. By utilizing Sankey and violin diagrams to illustrate the correlations between various risk groups and subtypes A and B, the objectivity and predictive precision of the model were confirmed. A heatmap was employed to illustrate the relationships between the expression of model genes, survival time, and risk scores.

We employed the CIBERSORT package to assess the relative abundance of 22 distinct immune cell types in each sample from the training set. Violin plots were then used to compare immune cell abundance levels between the high and low PCDS groups. Additionally, the R package estimate was utilized to evaluate potential differences in stromal scores, immune scores, and estimated scores within the tumor microenvironment between these two groups.

### 2.6 Evaluation of mRNA expression levels in model genes

Total RNA was isolated from the tissues or cells according to the manufacturer’s instructions using Trizol reagent (R401, Vazyme).The RNA’s quantity and integrity were then verified using a spectrophotometer. Subsequently, cDNA was synthesized from the RNA template employing reverse transcriptase and random primers. This cDNA served as the template for quantitative polymerase chain reaction (qPCR), conducted on a StepOnePlus Real-Time PCR System (ABI, USA), to measure the expression levels of the MLH1, SQLE, EDIL3, MTM1, and CLTCL1 genes. The qPCR procedure followed the manufacturer’s instructions, utilizing SYBR Green (Q712, Vazyme) and gene-specific primers. An internal control was utilized instead of beta-actin. Relative gene expression levels were determined using the 2^-ΔΔCT method. Details of the primers can be found in **Supplementary Table S2**.

### 2.7 Determination of protein expression levels of model genes

Protein extraction from tissue or cell lysates was performed using radioimmunoprecipitation assay (RIPA) buffer (abs9231, absin). The isolated proteins were subsequently subjected to sodium dodecyl sulfate-polyacrylamide gel electrophoresis (SDS-PAGE) and then transferred onto polyvinylidene fluoride (PVDF) membranes. After transfer, the membranes were incubated with primary antibodies against EDIL3 (ab190692, Abcam), GAPDH (ab9485, Abcam), CLTCL1 (ab21679, Abcam), and SQLE (ab67479, Abcam). Following this, a horseradish peroxidase (HRP)-conjugated secondary antibody (ab6721, Abcam) was applied. Protein bands were detected using an enhanced chemiluminescence (ECL) substrate (E411 Vazyme), which reacts with HRP to produce light, thereby visualizing the proteins on the membrane. GAPDH expression served as the internal control.

### 2.8 Culturing and transfection of cells

The osteosarcoma cell lines (U2OS, MG-63, and HOS) as well as mesenchymal stem cells (MSCs) were obtained from the American Type Culture Collection (ATCC). Each cell line was cultured and maintained following the specific protocols provided by their respective suppliers. For the osteosarcoma cell lines, Dulbecco’s Modified Eagle Medium (DMEM) (Gibco, USA) was used, while a specialized Human Mesenchymal Stem Cell Growth Medium (Gibco, USA) was utilized for the MSCs. The cells were then placed in a humidified incubator set at 37°C with 5% CO2 to ensure optimal growth conditions. Plasmid transfection was performed following the manufacturer’s guidelines using Lipofectamine 2000 reagent (cat#11668019, Invitrogen). Plasmids were synthesized by GenePharma.

### 2.9 Detection of proliferative capacity of osteosarcoma cells after knockdown of model gene expression

Cells were plated at a density of 5,000 cells per well in a 96-well plate and were given 24 hours to attach and acclimate. After this initial period, the cells were cultured for a standardized duration to ensure synchronization across the population. Subsequently, the CCK-8 assay reagent (C0038, Beyotime) was added to each well following the manufacturer’s instructions. The plate was incubated for an additional 120 minutes to allow the colorimetric reaction to develop. Cell viability was subsequently evaluated by measuring the absorbance at 450 nm with a microplate reader. The optical density readings were used as indicators of cell proliferation and overall viability.

For the colony formation assay, cells were detached with trypsin and evenly seeded into 6-well plates at a concentration of 1,000 cells per well. The cells were cultured for another 7 days to allow colony formation. Subsequently, the cell monolayers were fixed using a 4% solution of paraformaldehyde and then incubated with a 0.1% crystal violet staining solution (abs817172, absin) to visualize the colonies. Photographs of the stained colonies were captured, and the colony counts were documented and analyzed among the various experimental groups.

### 2.10 Detection of migration ability of osteosarcoma cells after knockdown model gene expression

When the cell monolayer reached around 90% confluence, a wound was created by gently scraping a specific area of the cell layer with a 200 μl plastic pipette tip, producing a controlled scratch. Images of the scratch wound were taken immediately after its creation and again after a 24-hour incubation period using a phase-contrast or inverted microscope equipped with a digital camera.

### 2.11 Detection of apoptosis rate of osteosarcoma cells after knockdown of model gene expression

The cells underwent a staining procedure that included a one-hour incubation with Annexin V-FITC (A211, Vazyme) and 7-AAD in the absence of light and at room temperature. After the designated incubation period, the cells underwent three comprehensive washes using cold phosphate-buffered saline (PBS) in order to eliminate any unbound stains. Following that, the cells were reconstituted in PBS, and a flow cytometer (BD, USA) was utilized to analyze the stained cells.

### 2.12 Single-cell analysis

Six samples of osteosarcoma (OS) and six samples of normal control make up the GSE162454 dataset. The R program Seurat was used to perform quality control processes for the single-cell sequencing data. The tSNE and UMAP algorithms were then used for dimension reduction and clustering, respectively. The generated two-dimensional graphs were labeled with numbers to identify cell types. Further annotation of cell types was performed using the singleR package in R to identify established cell types that are linked to each cellular subgroup.

### 2.13 Drug sensitivity analysis

Using the oncoPredict R package, sensitivity scores for 198 anticancer drugs were calculated for samples in the training set. Through iterative analysis, we identified significant sensitivity differences between the OS-PCDS high and OS-PCDS low groups, with a p-value threshold of <0.001. This analysis revealed significant sensitivity differences in 34 drugs: 9 drugs demonstrated increased sensitivity in the PCDS low group, while 25 drugs showed heightened sensitivity in the PCDS high group. The results of the drug sensitivity screening were visually represented using violin plots, highlighting the differences across the various risk categories.

### 2.14 Osteosarcoma specimen information

To assess the mRNA and protein expression levels of the model genes, we collected tumor specimens and adjacent normal tissue samples from six osteosarcoma patients. These samples were obtained during surgeries performed at the Affiliated Zhongshan Hospital of Dalian University between September 2021 and September 2023. Prior to specimen collection, informed assent was obtained from all patients. In addition, the research protocol, including the collection and use of human tissue samples, has been reviewed and approved by batch number KY2023-115-1 of the hospital’s ethical review committee.

### 2.15 Statistical analysis

Statistical analyses were performed using R software version 4.1.3. Continuous variables were presented as means with standard deviations or as medians with interquartile ranges, while categorical variables were summarized using frequencies and percentages. T-tests were utilized to compare continuous variables between two groups, and one-way ANOVA was applied for comparisons involving more than two groups. For categorical variables, chi-square tests or Fisher exact tests were used as appropriate to identify significant differences.

Survival distributions between different groups were compared using the Kaplan-Meier method along with the log-rank test. The Cox proportional hazards regression model was employed to evaluate the prognostic value of the osteosarcoma programmed cell death score (OS-PCDS), adjusting for potential confounding variables. Model performance was assessed using the concordance index (C-index), and receiver operating characteristic (ROC) curve analyses were conducted to calculate the area under the curve (AUC), thereby evaluating model effectiveness.

To optimize the model, machine learning algorithms such as Lasso, Ridge regression, and Random Survival Forests were employed. These algorithms were selected for their capability to handle high-dimensional data and perform feature selection effectively. Cross-validation was used for parameter tuning to avoid overfitting and ensure the model’s robustness.

A two-sided P-value of less than 0.05 was considered statistically significant. Detailed statistical analyses for specific experiments, including qPCR, CCK8 assays, flow cytometry, and other investigations, were performed using Prism software. For qPCR, gene expression levels were normalized to internal controls, and relative quantification was carried out using the 2^-ΔΔCT method. CCK8 assay results were analyzed by comparing optical density values across different time points and treatment groups using repeated measures ANOVA. Flow cytometry data were analyzed by calculating the percentage of cells in different cell cycle stages or the percentage of apoptotic cells, followed by statistical comparisons using T-tests.

## 3 Results

### 3.1 Identification of differentially expressed and prognosis-associated genes

**Figure 1:**
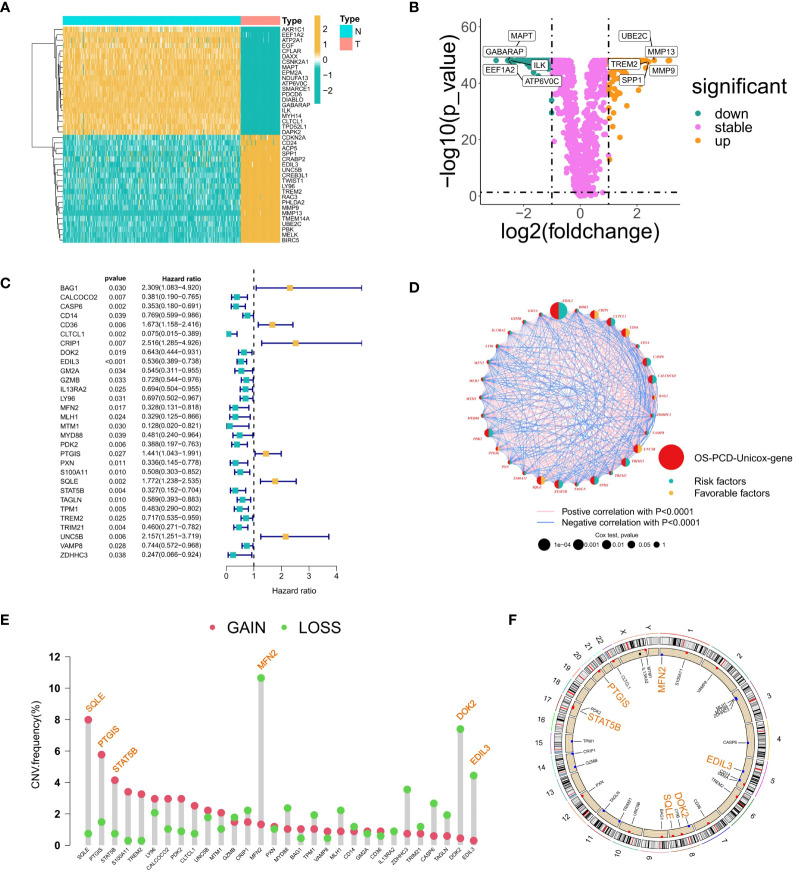
Gene Selection Relating to Prognosis and Differentiation. **(A)** Heatmap showing the expression patterns of 40 randomly selected differentially expressed genes between osteosarcoma samples and adjacent normal tissues. The color scale indicates the level of gene expression, with red representing high expression and blue representing low expression. **(B)** A volcano diagram illustrating the correlation between fold changes, P-values, and gene expression levels; yellow dots denote upregulated genes, green dots represent downregulated genes, and purple dots signify insignificant differences in gene expression. **(C)** A forest plot illustrating thirty genes that have been linked to survival prognosis. **(D)** A circos plot depicting the correlations among genes associated with prognosis. **(E)** A lollipop diagram illustrating the frequency of copy number variations (CNVs) in genes associated with prognosis, with particular attention given to CNVs surpassing a 4% threshold. **(F)** Genes with a mutation frequency greater than 4% are highlighted at the position of chromosome mutations. The genomic circumference diagram illustrates the chromosomal locations of genes that display high-frequency CNVs.

Within the training set, 246 genes were found to be differentially expressed between osteosarcoma samples and their corresponding adjacent normal tissues. Among these, 145 genes showed significant downregulation, while 101 genes demonstrated significant upregulation. A heatmap depicted the expression patterns of forty randomly selected genes (**Figure 1A**). The volcano plot illustrated both upregulated and downregulated genes, with notable upregulated genes including UBE2C, MMP13, TREM2, SPP1, and MMP9, and representative downregulated genes including MAPT, GABARAP, ILK, EEF1A2, and ATP6V0C (**Figure 1B**).

A total of 30 prognosis-related genes were identified and visualized using a forest plot. This group included 24 protective genes (such as CLTCL1, CALCOCO2, MLH1, MTM1, and ZDHC3) and 6 risk genes (including BAG1, CD36, and CRIP1) (**Figure 1C**). Additionally, a circos plot was utilized to illustrate the interconnections among these prognosis-associated genes. Genes related to prognosis were highlighted in red, while genes carrying protective or risk factors were depicted with yellow and green semicircles, respectively. The sizes of circles representing p-values from Cox regression analysis served as indicators of the relative importance and relevance of gene interactions within the network. Particularly noteworthy genes included EDIL3, CRIP1, CLTCL1, SQLE, PDK2, and TPM1, which exhibited extensive interaction patterns. Expression correlations between genes were represented by lines connecting them, with positive correlations depicted in pink and negative correlations in blue (**Figure 1D**).

A bar plot was created to display the frequency of Copy Number Variations (CNVs) impacting prognosis-associated genes, focusing on those with frequencies above 4%. Noteworthy genes with copy number gains included SQLE, PTGIS, and STAT5B, while those with losses comprised MFN2, DOK2, and EDIL3 (**Figure 1E**). Furthermore, a circular plot depicted the chromosomal locations of these high-frequency CNV-affected genes: MFN2 on chromosome 1, EDIL3 on chromosome 5, SQLE and DOK2 on chromosome 8, STAT5B on chromosome 17, and PTGIS on chromosome 20. Chromosomes 3, 5, 8, and 17, in particular, exhibited high frequencies of CNV events in osteosarcoma (**Figure 1F**).

### 3.2 Revealing the establishment and mechanism analysis of osteosarcoma subtypes

**Figure 2:**
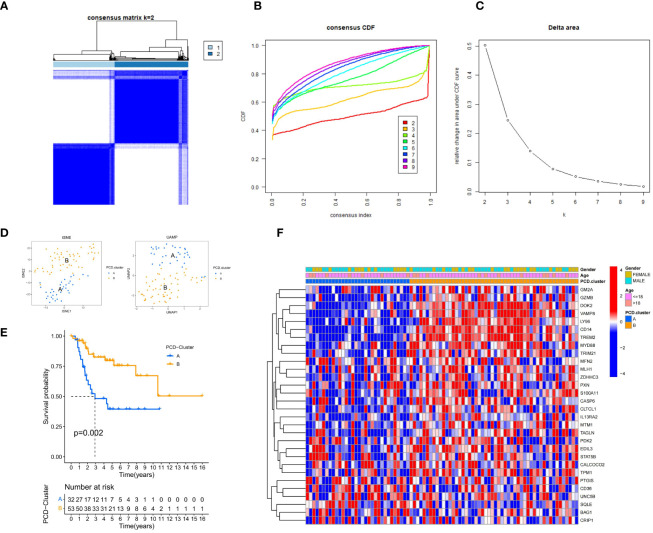
Clinical Information and Subtype Differentiation Are Illustrated in **Figure 2**. **(A)** By employing the R package ConsensusClusterPlus and non-parametric clustering, TARGET-OS samples were categorized into subtypes A and B. **(B)** The determination of the optimal number of clusters is based on the x-coordinate of the inflection point on the variability curve of the CDF. **(C)** Cluster number CDF curves spanning the range of 2 to 9. **(D)** The osteosarcoma expression matrix is reduced to two dimensions by the UMAP and tSNE algorithms, which distinguish the expression patterns of the two subtypes. **(E)** K-M survival analysis curve. The Kaplan-Meier diagram (P = 0.002) provides validation for the prognostic distinctions among the four subtypes. **(F)** A pheatmap presents the subtype and clinical information of each sample.

The ConsensusClusterPlus R package was employed to determine the optimal number of clusters for unsupervised clustering, categorizing TARGET-OS samples into subtypes A and B (**Figure 2A**). The optimal clustering number was identified by analyzing the inflection point on the variation rate curve of the Cumulative Distribution Function (CDF) (**Figures 2B, C**). A heatmap was generated to display the subtype and clinical information for each sample (**Figure 2F**). Additionally, dimensionality reduction of the osteosarcoma expression matrix to two dimensions was performed using the t-distributed Stochastic Neighbor Embedding (tSNE) and Uniform Manifold Approximation and Projection (UMAP) algorithms, with each sample assigned a point value (**Figure 2D**). Both algorithms effectively distinguished subtypes A and B, confirming the objectivity of PCD-related osteosarcoma subtyping. Kaplan-Meier (KM) curves further validated prognostic differences between the subtypes (P = 0.002), with subtype B showing better survival outcomes than subtype A (**Figure 2E**).

**Figure 3:**
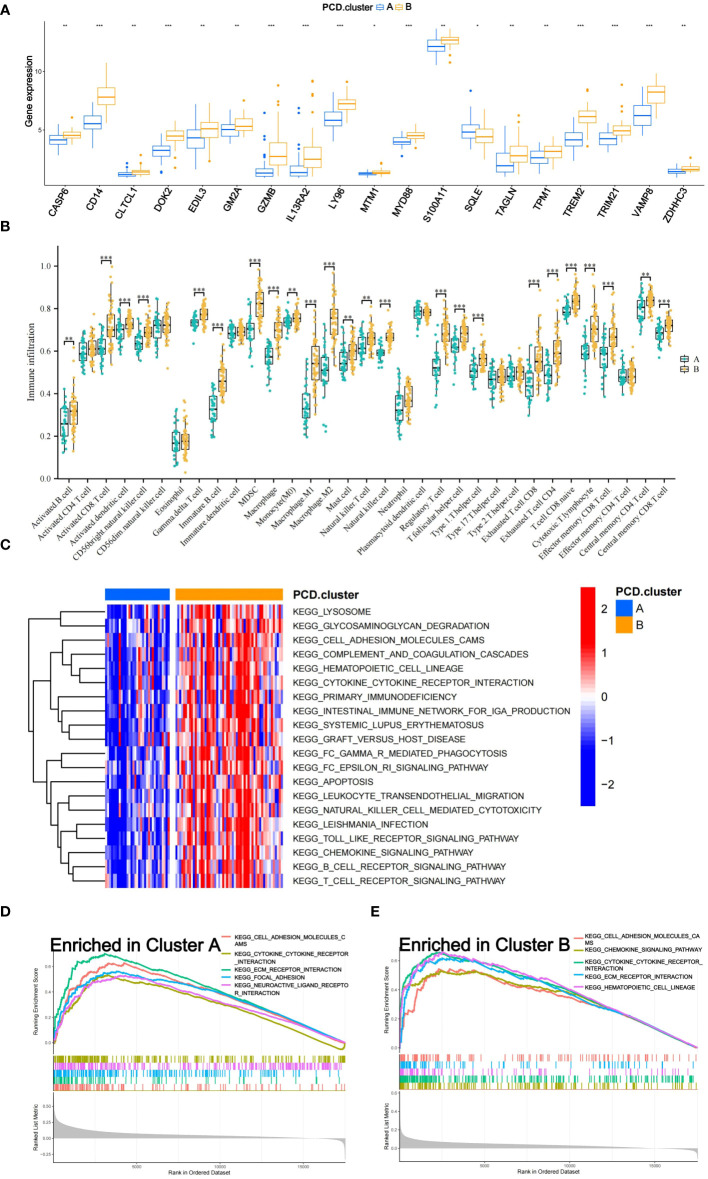
Mechanisms of Subtypes of Osteosarcoma Associated with PCD. **(A)** Box diagram depicting the variation in prognostic gene expression among distinct subtypes. **(B)** The differences in 33 immune cell abundances between the two subtypes are depicted in the box diagram. **(C)** The Gene Set Variation Analysis (GSVA) method was applied to identify pathways that varied in expression between two subtypes. **(D, E)** Gene Set Enrichment Analysis (GSEA) shows which five pathways within each subtype have the highest level of enrichment. The following p-value ranges: * < 0.05, ** < 0.01, and *** < 0.001.

An in-depth analysis was subsequently conducted to uncover the molecular mechanisms underlying the prognostic differences among the subtypes. The limma package facilitated differential expression analysis of prognosis-associated genes between subtypes, with results presented via box plots (**Figure 3A**). A total of 19 genes displayed significant differential expression between subtypes A and B, with 18 genes upregulated in subtype B and only one gene upregulated in subtype A. The ssGSEA algorithm computed the abundance of 33 distinct immune cell types in each training set sample. Box plots visualized the differences in immune cell expression levels between the subtypes (**Figure 3B**). Significant variations were observed in the abundance of 24 immune cell types, with all being more abundant in subtype B, except for nine cell types, including immature dendritic cells, eosinophils, Th17 cells, and Th2 cells.

The GSVA algorithm was utilized to examine differential enrichment pathways between the subtypes. A heatmap highlighted the top 20 pathways with significant enrichment differences, all upregulated in subtype B (**Figure 3C**). GSEA curves depicted the top five pathways most enriched in each subtype (**Figures 3D, E**). Subtype A showed significant upregulation in the Focal Adhesion and Neuroactive Ligand-Receptor Interaction pathways, while subtype B demonstrated substantial upregulation in the Hematopoietic Cell Lineage and Chemokine Signaling Pathway pathways. Additionally, three pathways—Cell Adhesion Molecules (CAMs), Cytokine-Cytokine Receptor Interaction, and Extracellular Matrix (ECM) Receptor Interaction—were notably upregulated in both subtypes.

### 3.3 Construction of machine learning integration models

**Figure 4:**
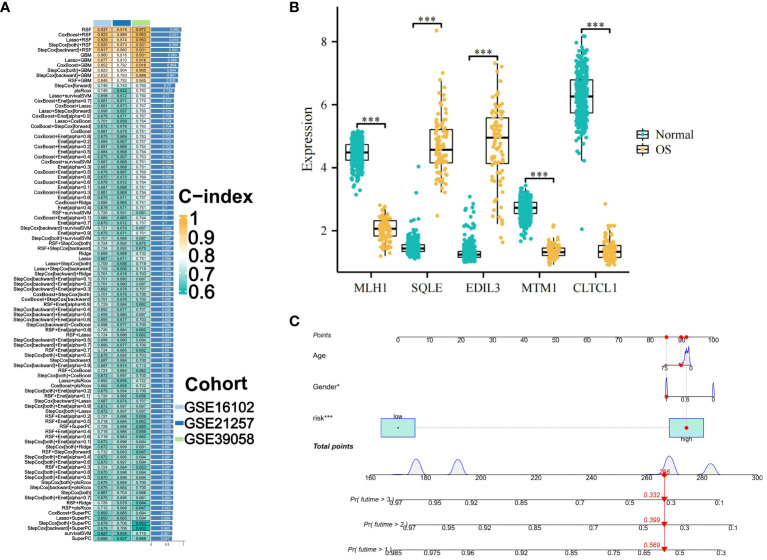
Construction and Selection of an Machine Learning Integration Model. **(A)** A cumulative sum of 101 iterations generated by ten distinct machine learning algorithms was employed in the development of prognostic models, with the Concordance index (C-index) being computed. The optimal model was determined to be the RSF algorithm, and its score was designated OS-PCDS (Osteosarcoma Programmed Cell Death Score). **(B)** The OS-PCDS gene expression levels in adjacent normal tissues and osteosarcoma are depicted in the box plot. **(C)** A nomogram model was developed by incorporating PCDS, gender and age variables. P values for *** is less than 0.001.

The integration of the machine learning algorithm generated a total of 101 possible combinations. The optimal model was identified to be the one constructed by the RSF algorithm, which exhibited the highest average consistency index and relatively low variability in the consistency index. This model’s scoring system was termed OS-PCDS. The model generated by the RSF algorithm ranked highest across all three GEO validation sets, with an average C-index of 0.943 (**Figure 4A**). During the variable selection process, the RSF algorithm identified all five prognostic-related genes, namely MTM1, MLH1, CLTCL1, EDIL3, and SQLE. The mRNA expression levels of these five genes showed significant differences between osteosarcoma samples and adjacent normal tissues, as depicted in the boxplots (**Figure 4B**). Specifically, EDIL3 and SQLE exhibited significant upregulation in osteosarcoma samples, suggesting their roles in promoting angiogenesis and cholesterol biosynthesis, respectively. EDIL3, known for its involvement in endothelial cell adhesion, may facilitate tumor angiogenesis, while SQLE, an enzyme in the cholesterol biosynthesis pathway, could support membrane biogenesis in rapidly proliferating tumor cells. While MTM1, MLH1, and CLTCL1 showed significant upregulation in adjacent normal tissues. By integrating clinical information from all datasets with the OS-PCDS, a nomogram model was developed to predict the prognosis of OS patients (**Figure 4C**).

### 3.4 Validation of machine learning integration models

**Figure 5:**
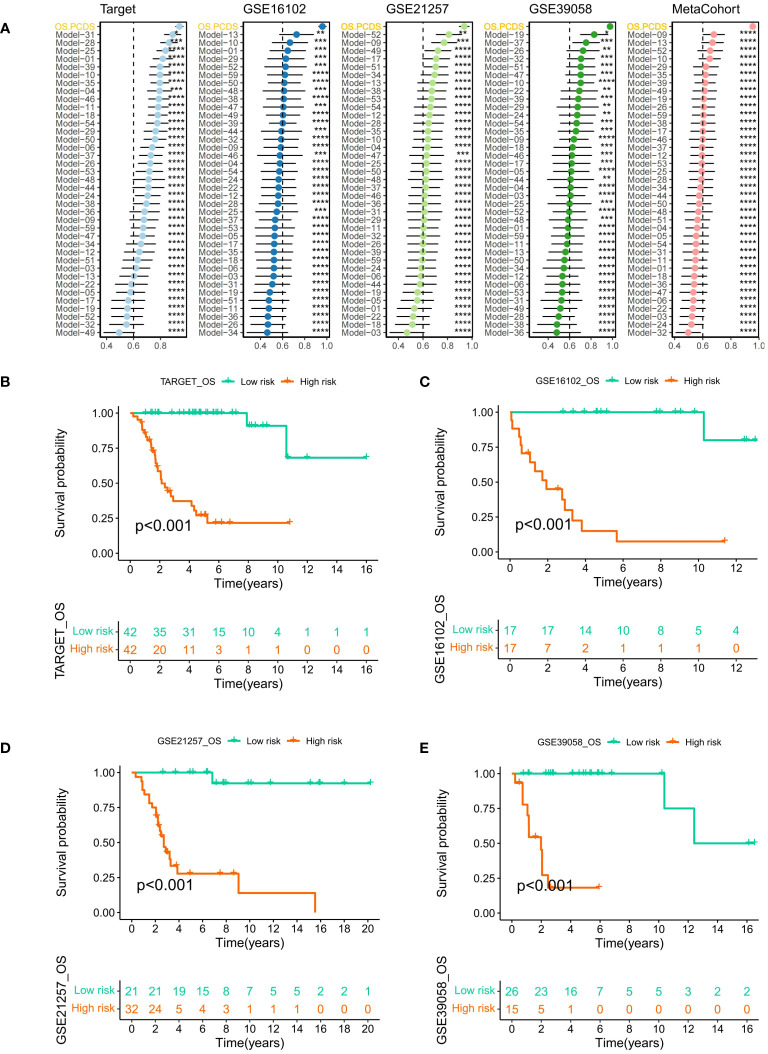
**(A)** A forest plot illustrating the OS-PCDS’s Superiority in published osteosarcoma models according to the C-index. Calculations were done for the TARGET-OS training set, several GEO validation sets, and a meta-cohort consisting of the GEO validation sets. **(B–E)** The median PCDS of the TARGET–OS training set was used as the cut-off value to separate all data into PCDS–high and PCDS–low categories. After that, Kaplan-Meier plot was generated for each dataset. Various degrees of importance are indicated by the following symbols: P values for *, **, ***, and **** are less than 0.05, 0.01, 0.001, and 0.0001, respectively.

A comparative analysis was conducted on osteosarcoma prognostic models published in the last five years. The C-index for both training and validation sets was illustrated using forest plots, with significant differences indicated by asterisks. Across all three GEO validation sets, the TARGET-OS training set, and the combined meta-cohort from the three validation sets, OS-PCDS consistently ranked highest in C-index values (**Figure 5A**). Samples were classified as high-risk or low-risk based on the median OS-PCDS value from the training set. Kaplan-Meier plots for each dataset showed significant survival differences (p < 0.05) between high-risk and low-risk groups in both the training set and the three validation sets. The prognosis of the low-risk group consistently surpassed that of the high-risk group in all datasets (**Figures 5B–E**).

**Figure 6:**
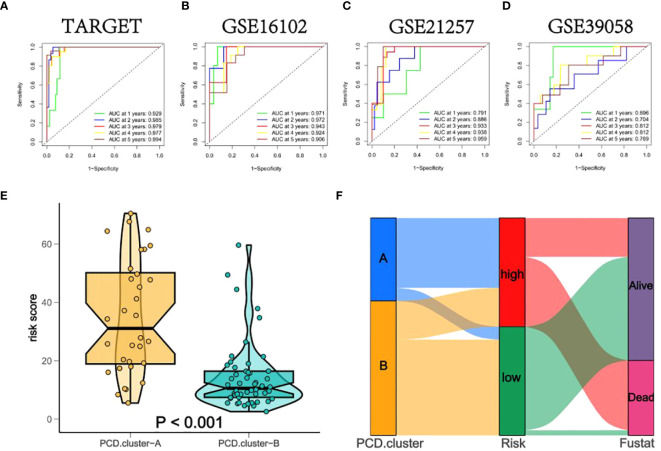
**(A–D)** The Area Under the Curve (AUC) values of PCDS at 1, 2, 3, 4, and 5 years for all training and validation sets. **(E)** Box diagrams indicate that the risk scores for subtypes A and B differ significantly. **(F)** A Sankey plot depicting noteworthy correlations between the sample distributions of the PCD-Cluster and OS-PCDS.

In both training and validation sets, the Area Under the Curve (AUC) values for PCDS at 1, 2, 3, 4, and 5 years were calculated using Receiver Operating Characteristic (ROC) curves. The 5-year AUC values for the TARGET, GSE16102, GSE21257, and GSE39058 cohorts were 0.994, 0.906, 0.959, and 0.769 respectively (**Figures 6A–D**), indicating the model’s exceptional stability. Violin plots displayed significant differences in OS-PCDS risk scores between subtypes A and B. Subtype B had lower risk scores associated with better prognostic outcomes, whereas subtype A had higher risk scores linked to poorer survival (**Figure 6E**). Additionally, the Sankey plot (**Figure 6F**) showed a significant correlation between the distribution of high-risk and low-risk categories and the A and B subtypes.

**Figure 7:**
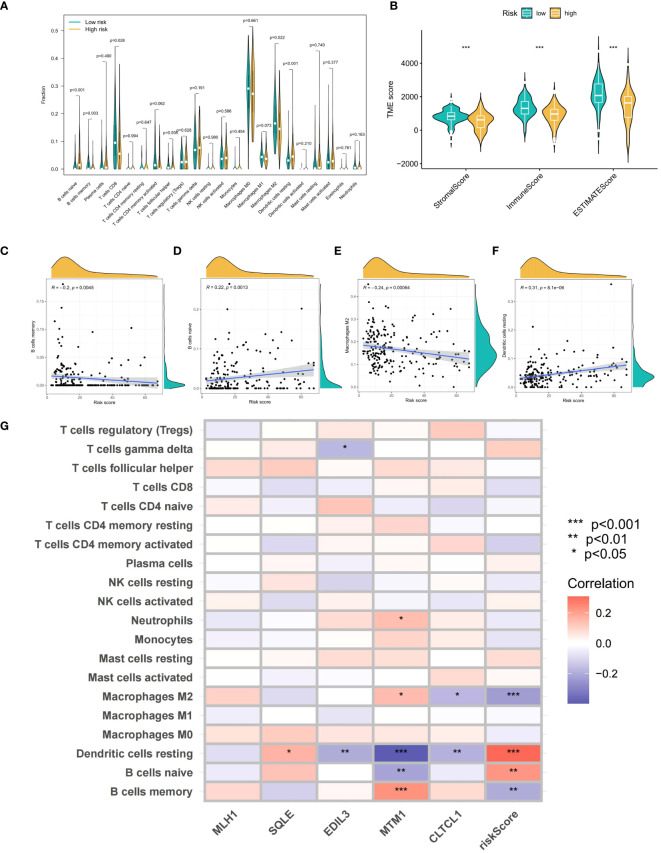
**(A)** shows violin plots generated by the CIBERSORT algorithm to compare the quantity of 22 immune cell types across groups with high and low scores. **(B)** Violin graphs showing statistically significant differences in stromal and estimate scores between PCDS-high and PCDS-low groups are produced using estimate analysis. **(C–F)** Correlation scatter graphs show how PCDS is related to four different kinds of immune cells. **(G)** A heatmap showing the correlation between immune cell abundance and five model gene expression levels. The following is a notation for the importance levels: The following p-value ranges: * < 0.05, ** < 0.01, and *** < 0.001. P-values are considered statistically significant when they are less than 0.05.

Using ESTIMATE analysis, violin plots were created to depict significant differences in stromal and immune scores between the high and low OS-PCDS groups (**Figure 7B**). The CIBERSORT algorithm was then applied to determine the abundance of 22 different immune cell types in each sample (**Figure 7A**). Violin plots revealed an increase in memory B cells and macrophage M2 cells in the low OS-PCDS group, while the high OS-PCDS group had elevated levels of naive B cells and resting dendritic cells. Correlation scatter plots further validated the relationship between these immune cells and OS-PCDS (**Figures 7C–F**). Additionally, a heatmap was generated to show the correlation between the abundance of immune cells and the expression levels of the five model genes, with significance levels indicated as * p < 0.05, ** p < 0.01, *** p < 0.001 (**Figure 7G**).

### 3.5 The mRNA expression levels of the model genes were evaluated

**Figure 8:**
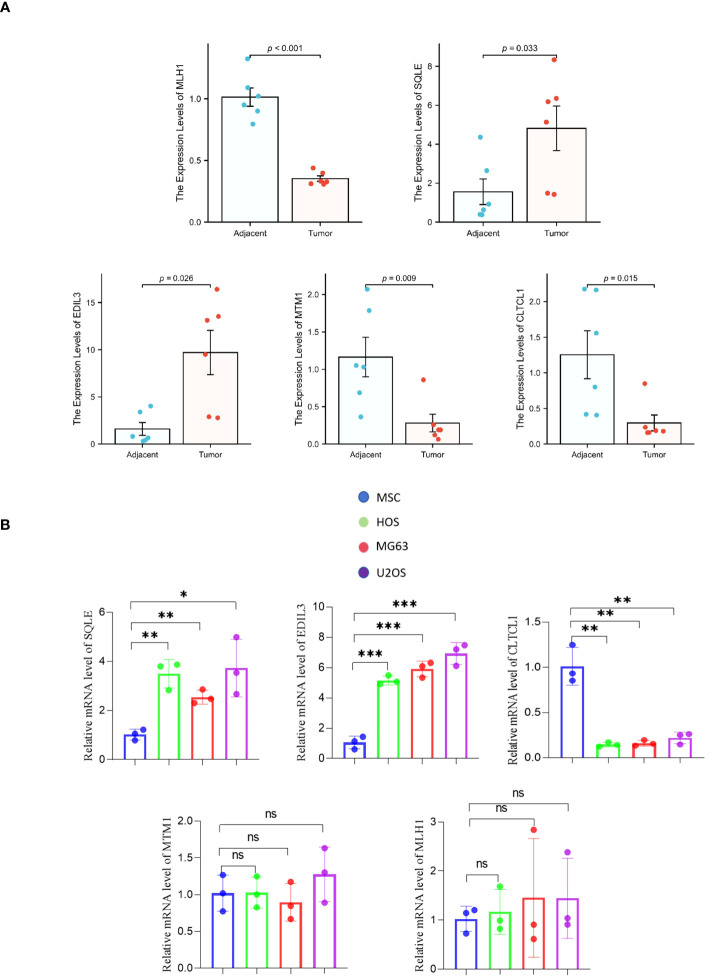
**(A)** The differential mRNA expression of the model gene between osteosarcoma samples and adjacent normal tissues. **(B)** A comparison is made between human osteosarcoma cell lines (HOS, MG63, U2OS) and mesenchymal stem cell (MSC) cell lines with respect to the mRNA expression of the model gene. The levels of significance are denoted as follows: *pvalue < 0.05, **pvalue < 0.01; ***pvalue < 0.001. P-values that are below 0.05 are deemed to be statistically significant.

In comparison to osteosarcoma samples, the mRNA expression levels of the CLTCL1, MTM1, and MLH1 genes were significantly elevated in adjacent normal tissues (P=0.015, P=0.009, and P<0.001, respectively). On the other hand, the expression levels of SQLE and EDIL3 genes were considerably higher in osteosarcoma samples compared to the adjacent normal tissues (P=0.033 and P=0.026, respectively) (**Figure 8A**).

Quantitative polymerase chain reaction (qPCR) analysis of model genes in Mesenchymal Stem Cells (MSCs) and three different osteosarcoma cell lines (HOS, MG63, and U2OS) showed no significant differences in the expression levels of MLH1 and MTM1 genes between MSCs and the osteosarcoma cell lines. However, mRNA expression levels of EDIL3 and SQLE genes were markedly higher in osteosarcoma cell lines compared to MSCs. Furthermore, the CLTCL1 gene expression was significantly greater in MSCs than in the osteosarcoma cell lines (**Figure 8B**).

### 3.6 Expression levels of model genes encoding proteins

**Figure 9:**
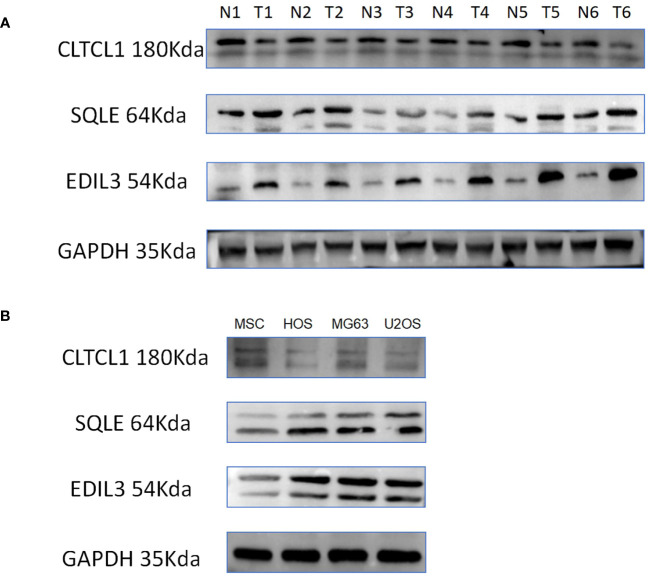
**(A)** The differences in the expression levels of CLTCL1, EDIL3, and SQLE proteins in 6 pairs of osteosarcomas and their adjacent normal tissues were demonstrated, with N representing normal tissues, T representing osteosarcoma tissues, and GAPDH protein being the internal reference protein. **(B)** The discrepancy between human osteosarcoma cell lines (HOS, MG63, U2OS) and MSC (mesenchymal stem cell) cell lines in terms of protein expression of the model gene is illustrated.

In adjacent normal tissues, the protein expression levels of CLTCL1 were significantly higher than those observed in osteosarcoma samples. Conversely, the protein expression levels of SQLE and EDIL3 were markedly elevated in osteosarcoma samples compared to the adjacent normal tissues (**Figure 9A**). Additionally, when compared to MSC cell lines, human osteosarcoma cell lines showed significantly higher protein expression levels of SQLE and EDIL3, whereas CLTCL1 demonstrated significantly higher protein expression levels in MSC cell lines than in human osteosarcoma cell lines (**Figure 9B**).

### 3.7 Impact of gene knockdown on osteosarcoma cell proliferative capacity

**Figure 10:**
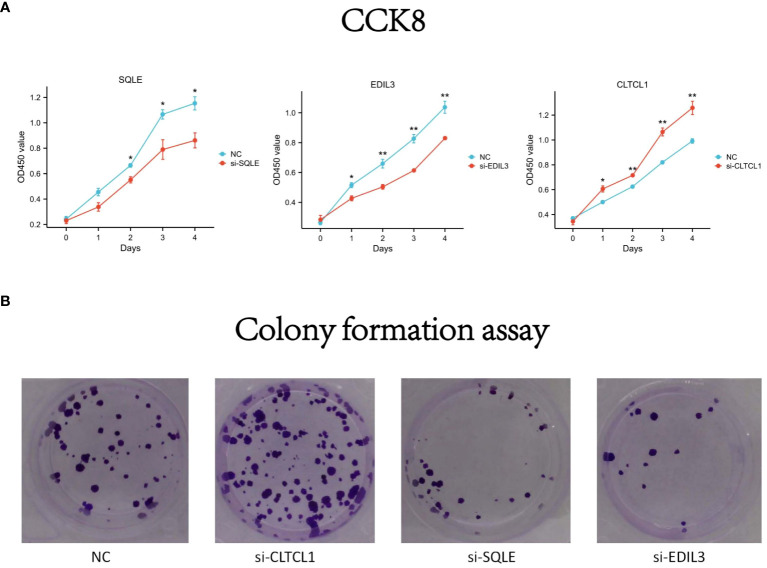
**(A)** The differences in optical density (OD) values over time compared to the negative control (NC) following knockdown of CLTCL1, SQLE, and EDIL3 gene expression levels in the CCK8 experiment. **(B)** The results of a colony formation assay. Four groups are depicted: negative control (NC), and cells treated with small interfering RNA (siRNA) against CLTCL1 (si-CLTCL1), SQLE (si-SQLE), and EDIL3 (si-EDIL3), respectively. The plates have been stained to visualize the colonies. Significance levels are indicated as *p value < 0.05, **p value < 0.01 ***p value < 0.001. P-values less than 0.05 are considered statistically significant.

To evaluate the differential expression of CLTCL1, SQLE, and EDIL3 genes in OS cells (U2OS), individual gene silencing was conducted, and the differences in proliferation capacity between the silenced and control groups were assessed. In the CCK8 assay, the optical density (OD) values for the si-CLTCL1 group significantly increased from day one, showing a notable contrast to the control group. Conversely, the OD values for the si-SQLE group significantly decreased from day two, demonstrating a marked difference from the control. Similarly, the si-EDIL3 group exhibited a significant reduction in OD values compared to the control group from day one onward (**Figure 10A**).

Results from the colony formation assay showed a significant increase in the number of OS cell clones in the CLTCL1 knockdown group compared to the control. In contrast, there was a substantial decrease in the number of OS cell clones in the SQLE knockdown group. Additionally, the EDIL3 knockdown group also displayed a reduction in OS cell clone numbers (**Figure 10B**).

### 3.8 Repercussions of gene knockdown on the migratory capacity of osteosarcoma cells

**Figure 11:**
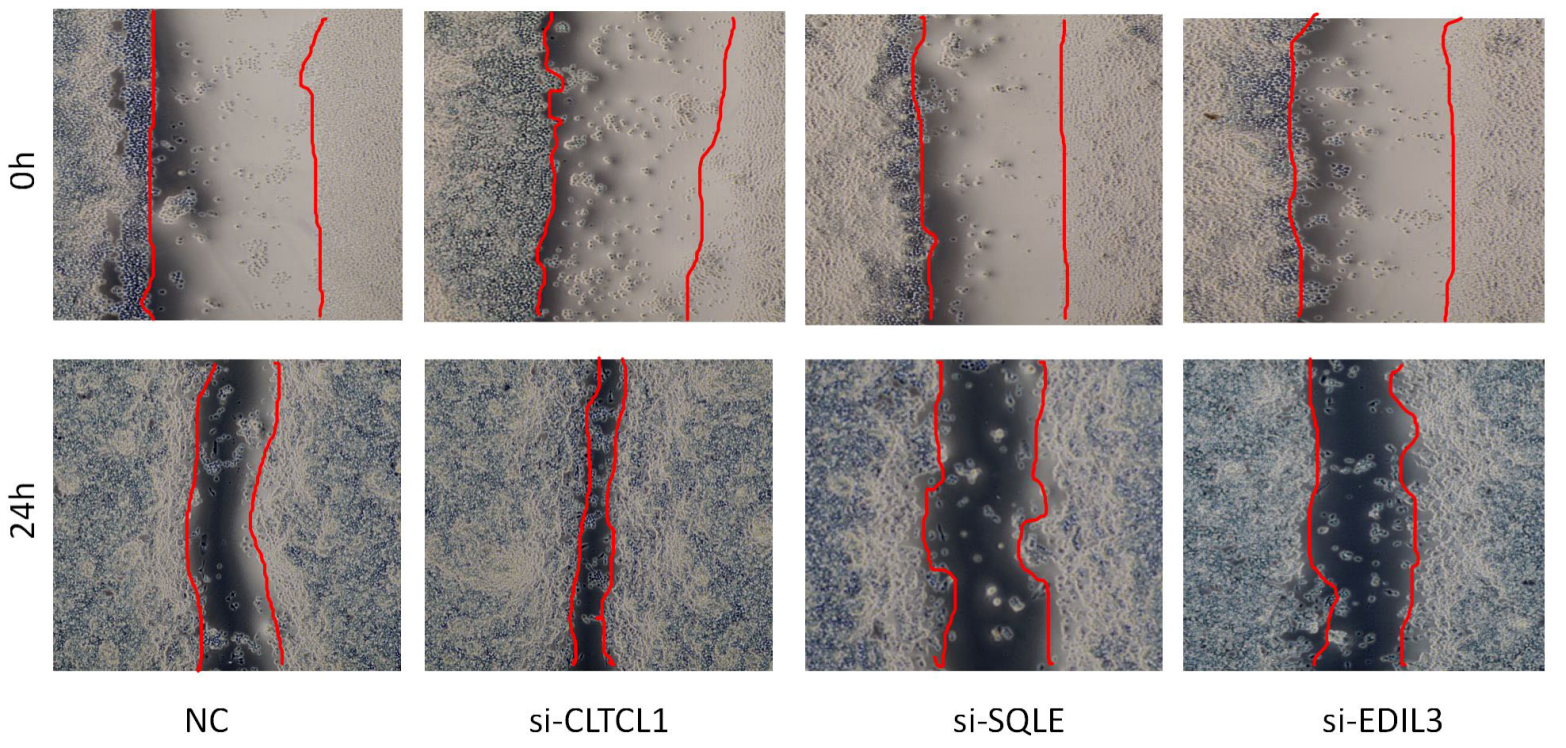
The image depicts a wound healing assay conducted to evaluate cell migration over a 24-hour period.

The results of the cell scratch assay revealed that, compared to the 0-hour time point, all four cell groups exhibited healing after 24 hours. Additionally, OS cells treated with CLTCL1 gene knockdown showed significantly enhanced migratory capacity compared to the control group, while those treated with SQLE and EDIL3 gene knockdown demonstrated weakened migratory abilities (**Figure 11**).

### 3.9 Repercussions of knockdown model genes on the apoptotic rate of osteosarcoma cells

**Figure 12:**
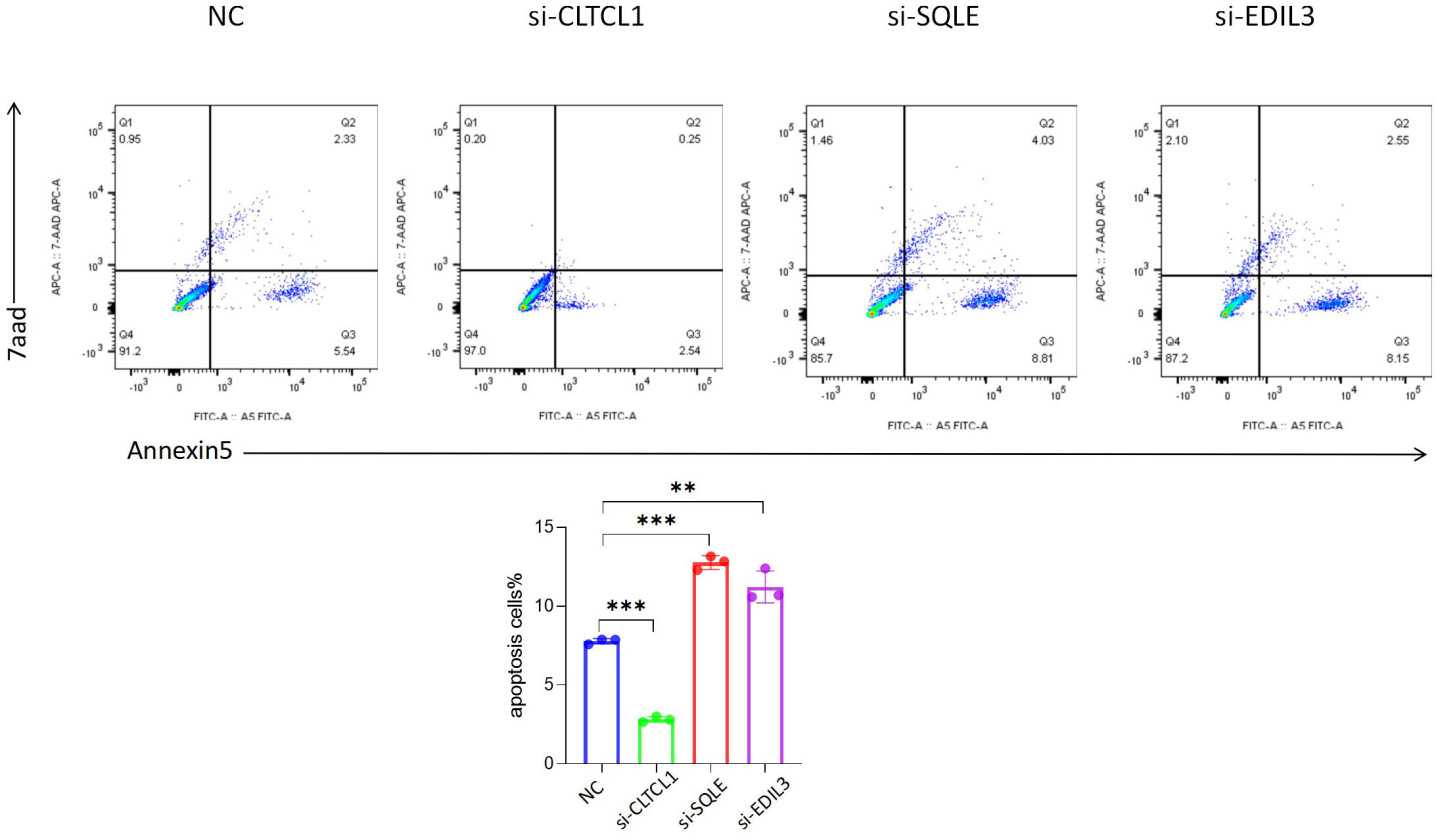
The results showed that compared with the negative control (NC), the apoptosis rate of osteosarcoma cells was changed after the expression levels of CLTCL1, SQLE and EDIL3 were reduced. Significance levels are indicated as *p value < 0.05, **p value < 0.01 ***p value < 0.001. P-values less than 0.05 are considered statistically significant.

The bar chart below depicts the comparison of apoptosis rates among different groups (**Figure 12**). Compared to the control group, a significant decrease in the apoptosis rate of osteosarcoma cells was observed following CLTCL1 gene knockdown (P<0.001), while a substantial increase was noted after SQLE gene knockdown (P<0.001) and EDIL3 gene knockdown (P<0.01).

### 3.10 Single-cell analysis

**Figure 13:**
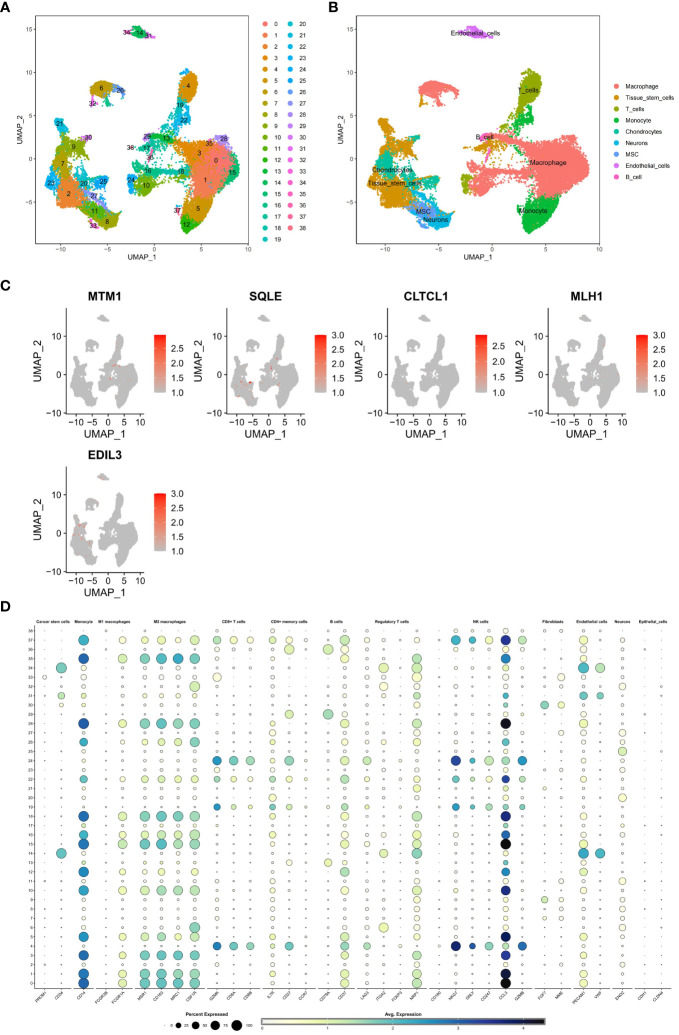
**(A, B)** A single-cell analysis was conducted on the GSE162454 dataset in order to examine the processes of dimensionality reduction and clustering of single-cell data. **(C)** To visually represent the expression levels of critical genes in various cell subgroups within the model, UMAP plots were employed. The dot plot illustrated the relationship between subgroups of cells. **(D)** The bubble map illustrates the expression levels of key genes in different cell subpopulations.

The GSE162454 dataset, which includes six osteosarcoma samples, underwent various analyses, and the results were compared to ensure validity. Cell subclusters identified through Seurat dimensionality reduction clustering and annotated known cell types from singleR were visually represented using UMAP plots (**Figures 13A, B**). Bubble plots depicted the correspondence between cell subclusters (**Figure 13D**). Furthermore, UMAP and violin plots were utilized to illustrate the expression levels of model hub genes within individual cell subclusters (**Figure 13C**). A notable finding was the significant upregulation of EDIL3 expression, particularly in chondrocytes and tissue stem cells.

### 3.11 Identification of OS-PCDS-related anticancer medications

**Figure 14:**
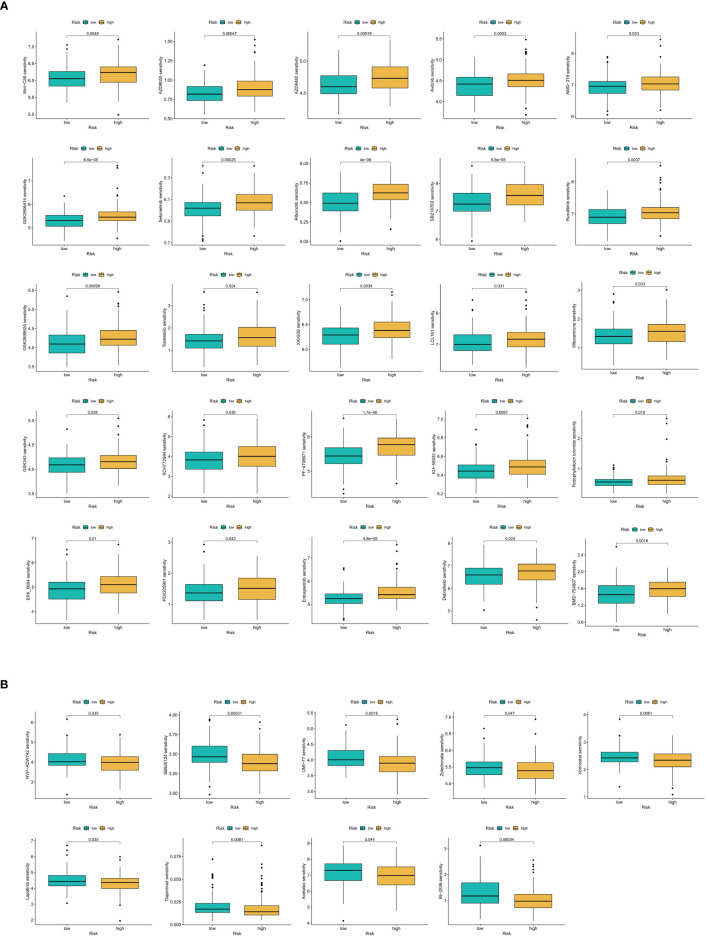
**(A)** Anticancer drug screening in relation to model scores. Twenty-five compounds demonstrate increased sensitivity in the OS-PCDS-high group. **(B)** Nine drugs show greater sensitivity in the OS-PCDS-low group. P-values less than 0.001 are considered statistically significant.

The violin plot clearly illustrates substantial differences in drug sensitivity between the high and low PCDS groups, highlighting a set of 34 drugs with divergent responses. Specifically, eight drugs, including Acetalax, BI-2536, Daporinad, and Lapatinib, showed increased sensitivity in the low PCDS group (**Figure 14A**). In contrast, 26 drugs, such as Axitinib, Dabrafenib, Entospletinib, and Mitoxantrone, exhibited enhanced sensitivity in the high PCDS group (**Figure 14B**).

## 4 Discussion

Osteosarcoma is a common and highly aggressive bone cancer that predominantly affects individuals during their teenage years (29).Despite progress in surgical techniques and chemotherapy, the 5-year survival rate for osteosarcoma patients has seen little improvement over the last twenty years (30, 31).The pathogenesis of osteosarcoma is exceedingly intricate, with single genes, protein markers, or conventional clinical data falling short in fully elucidating its complexity (32). These approaches are relatively rudimentary and overlook the heterogeneity inherent in osteosarcoma. Studies indicate that markers like B7-H3, GD2, and HER2, while closely linked to osteosarcoma pathogenesis, offer limited prognostic value when examined in isolation (33). As recent literature underscores, integrating molecular data is pivotal for enhancing prognosis (34). Traditional multigene models have been found lacking in exploring the interplay of genetic information in osteosarcoma. They overlook gene interactions and pathway crosstalk, thus constraining their predictive capability (35). Recent research endeavors have aimed to employ multi-gene panels in osteosarcoma, emphasizing the imperative for more intricate and comprehensive investigations into the disease (36, 37). The fifteen forms of programmed cell death play a crucial role in cancer (38). Therefore, this study screened genes highly correlated with programmed cell death and osteosarcoma, unveiling numerous potential therapeutic targets, pathways, and immune cells. By leveraging highly sophisticated machine learning integration, a prognostic model for osteosarcoma was constructed to furnish a more precise prognosis assessment for osteosarcoma patients.

Using unsupervised clustering, the osteosarcoma (OS) patient database was classified into A and B subtypes, revealing a poorer survival prognosis in subtype A. There were 19 genes with significant differential expression between the two subtypes, among which only one gene, SQLE, was highly expressed in subtype A. Previous research has demonstrated that the p53 transcription regulator can inhibit tumor cell growth through suppression of SQLE expression (39). Meanwhile, research has confirmed the role of CASP6, a gene highly expressed in subtype B, in reducing the risk of osteosarcoma patient prognosis (40). There was significant variation in immune cell infiltration abundance between the two subtypes. While 9 out of 33 immune cell types showed no significant distribution difference between subtypes, the remaining 24 immune cell types were significantly more abundant in subtype B. This suggests that the low abundance of immune cell infiltration is one of the factors contributing to the poorer survival prognosis in subtype A patients. Pathway enrichment analysis revealed differences between the two subtypes. Subtype A exhibited significant enrichment in the focal adhesion pathway, a finding consistent with previous studies that have associated this pathway with increased cancer cell growth and migration (41). In summary, by studying differences in survival prognosis between different subtypes, immune cell abundance, differential gene expression, and enriched pathways, we explore key factors leading to different survival prognoses in OS patients, providing a foundation for subsequent research on molecular targeted therapy for osteosarcoma.

In recent years, various prognostic models for osteosarcoma have been developed, mostly constructed using single machine learning algorithms and single forms of cell death to build prognostic risk models for osteosarcoma (42–44). However, the molecular characteristics of osteosarcoma are extremely complex, and using single screening criteria cannot accurately predict the prognosis of osteosarcoma patients. A study utilized multiple machine learning integration to construct a prognostic model for ovarian cancer and established a prognostic model for breast cancer patients using various forms of cell death (45, 46). However, these methods have not been applied to osteosarcoma disease. Samples of osteosarcoma patients and 15 sets of genes associated with PCD were extracted from the database. Prior to filtering out genes associated with prognosis, genes differentially expressed between osteosarcoma samples and adjacent normal tissues were screened. These genes were then intersected with PCD genes. The model utilized in this investigation was constructed using this gene set as its foundation.

As demonstrated in the text, our study’s machine learning integration model exhibits significant improvements compared to traditional methods. The use of multiple integrated machine learning algorithms resulted in improved accuracy of prognostic predictions for osteosarcoma, as evidenced by higher concordance index scores compared to traditional methods. The experimental results are consistent with findings from other studies, which have used similar methodologies and analytical frameworks (47–50). OS samples from all datasets are classified by the OS-PCDS into high-risk and low-risk groups, with survival analysis indicating that a lower prognosis is associated with the low-risk group and a higher prognosis is associated with the high-risk group. ROC curve analysis further validates the robustness of OS-PCDS. After undergoing internal validation, the predictive performance of OS-PCDS was compared to that of 38 other prognostic models for osteosarcoma based on transcriptomes that were published within the last five years. In order to mitigate discrepancies that may have emerged from sources other than the TARGET and GEO databases, all models underwent training and validation using homogeneous datasets. The results demonstrate that our model exhibits stronger stability and accuracy compared to existing prognostic tools, thus confirming its significant advantage and potential utility in clinical practice.

The machine learning integration selected five model genes: MLH1, MTM1, CLTCL1, EDIL3, and SQLE. In recent years, the roles of MLH1 and SQLE genes in osteosarcoma have garnered attention. Recent studies have indicated an association between the expression level of MLH1 and the prognosis of osteosarcoma patients. Specifically, the expression level of MLH1 shows a negative correlation with patients’ risk scores, suggesting that higher MLH1 expression is associated with lower risk scores and better prognosis (51). Kun reported that SQLE can promote the proliferation and migration of osteosarcoma cells (52). These studies suggest that MLH1 may act as a protective factor, with higher expression potentially reducing the risk and improving the prognosis of osteosarcoma patients, while SQLE may serve as a risk factor, with its higher expression possibly increasing the prognosis risk for osteosarcoma patients. Visualization of the expression levels of model genes in osteosarcoma samples and adjacent normal tissues from databases revealed that MLH1 was downregulated in osteosarcoma samples but significantly upregulated in adjacent normal tissues, whereas SQLE was significantly upregulated in osteosarcoma samples but downregulated in adjacent normal tissues. The expression patterns of MLH1 and SQLE suggest differential roles in osteosarcoma pathology. MLH1 was downregulated in osteosarcoma samples, potentially indicating a tumor suppressive function, whereas SQLE was upregulated, suggesting a role in tumor progression. The experimental results align with the conclusions of previous studies, suggesting that MLH1 and SQLE genes could serve as biomarkers for osteosarcoma prognosis and therapeutic targets. However, the precise mechanisms by which these genes influence osteosarcoma development remain to be fully elucidated. Various cellular experiments were conducted to elucidate the functional roles of the identified genes and their impacts on osteosarcoma cells. Specifically, the mRNA and protein expression levels of CLTCL1 were significantly reduced in osteosarcoma cells, indicating its potential role as a tumor suppressor gene. Bioinformatics analysis suggested that CLTCL1 might exert protective effects in osteosarcoma. Inhibition of CLTCL1 expression led to increased proliferation and migration of osteosarcoma cells, along with a significant reduction in apoptosis, validating its role as a protective molecular marker.

Further investigation into the regulatory mechanisms of risk genes, such as EDIL3 and SQLE, revealed that these genes are potential candidates for therapeutic targeting, as indicated by their significant role in the progression and prognosis of osteosarcoma. Downregulation of EDIL3 and SQLE in the U2OS osteosarcoma cell line was essential for exploring potential treatment strategies and understanding osteosarcoma pathology. Bioinformatics analysis indicated a positive correlation between the high expression of SQLE and EDIL3 and osteosarcoma cell development. Knockdown experiments showed that reduced expression of these genes led to a significant decrease in proliferation and migration abilities, along with an increase in apoptosis rates, suggesting their roles as risk factors that promote tumor development.

Additionally, the differential expression analysis using the limma package and subsequent gene set enrichment analyses (GSEA and GSVA) provided insights into the functional pathways and regulatory networks involving these differentially expressed genes. For instance, the upregulation of genes in the focal adhesion pathway in subtype A may promote cancer cell growth and migration, while the enriched immune-related pathways in subtype B indicate potential immunogenic responses. The mechanistic insights from these analyses highlight the complex interplay between gene expression patterns and osteosarcoma progression, emphasizing the need for further research to uncover the underlying biological mechanisms and therapeutic implications.

By integrating these findings, we can better understand the biological significance of the differentially expressed genes and their roles in osteosarcoma development and progression. This comprehensive approach lays the groundwork for future investigations into targeted therapies and prognostic biomarkers in osteosarcoma, enhancing the potential for personalized treatment strategies.

Risk genes are often utilized in the development of therapeutic targets. In our research, EDIL3 and SQLE may also serve as potential therapeutic targets. Therefore, we validated their functions by downregulating their expression levels in the human osteosarcoma cell line U2OS. This approach is essential for investigating potential treatment strategies and advancing our understanding of osteosarcoma pathology. In osteosarcoma cells, both the mRNA and protein expression levels of SQLE and EDIL3 genes show significant elevation. Combining the bioinformatics analysis results from this study, SQLE and EDIL3 are likely to be genes associated with risk, indicating a positive correlation between their high expression and the development of osteosarcoma cells. Knockdown of SQLE and EDIL3 gene expression in osteosarcoma cells resulted in a significant decrease in proliferation and migration abilities, accompanied by a notable increase in apoptosis rate. These findings suggest that SQLE and EDIL3 genes serve as risk factors in osteosarcoma, promoting tumor development.

In osteosarcoma cells, the mRNA expression levels of MTM1 and MLH1 genes were significantly decreased, suggesting their functions may be inhibited in these cells. Although further experimental validation of these two genes was not conducted in this study, combining the predictive results suggests that MTM1 and MLH1 genes are likely negatively correlated with tumor development. This conclusion awaits further experimental investigation.

While the prognostic model for osteosarcoma developed in this study demonstrates remarkably high predictive accuracy, it is imperative to acknowledge the inherent limitations of this research methodology. One significant constraint lies in the retrospective nature of the osteosarcoma patient data utilized, which were primarily sourced from the TARGET and GEO databases. This reliance on retrospective data may limit the generalizability of the findings to broader clinical contexts. Additionally, the variability in patient demographics and treatment protocols across different datasets could introduce biases that affect the model’s performance.

To conclusively ascertain the efficacy and robustness of the prognostic model, further validation through prospective clinical trials is essential. These trials would provide more controlled and comprehensive data, allowing for a more accurate assessment of the model’s predictive capabilities. Moreover, prospective studies could help in understanding how the model performs across diverse patient populations and in different clinical settings, thereby enhancing its applicability and reliability.

Recognizing these limitations aids in a fair assessment of the study’s scope and practicality, thereby furnishing crucial insights for subsequent inquiries. Further exploration of the molecular mechanisms governed by the genes identified in the model may unveil previously undiscovered therapeutic targets, potentially advancing more effective approaches to treating osteosarcoma. For instance, investigating the regulatory pathways and interactions of these genes could provide deeper insights into their roles in tumor progression and response to treatment. This understanding could lead to the development of targeted therapies that are more precise and effective in managing osteosarcoma.

In summary, while the current study provides a robust framework for prognostic modeling in osteosarcoma, addressing its limitations through future research and validation efforts is crucial for translating these findings into clinical practice. Continuous refinement and validation of the model will ensure its long-term utility and effectiveness in improving patient outcomes.

## 5 Conclusion

This study integrated multiple machine learning algorithms and selected five model genes, including CLTCL1, SQLE, EDIL3, MTM1, and MLH1. Utilizing these five genes, a programmed cell death-related osteosarcoma prognostic risk model was constructed, enabling a more precise and comprehensive analysis of prognostic factors in osteosarcoma patients. According to all experimental results, CLTCL1, MTM1, and MLH1 are likely tumor suppressor genes exerting inhibitory effects on osteosarcoma development, while SQLE and EDIL3 may function as targets promoting tumor proliferation, thus presenting new therapeutic potential. Further research, including *in vivo* studies and clinical trials, is crucial for validating the roles of these genes in osteosarcoma progression and assessing their potential as therapeutic targets.

## Data availability statement

The datasets presented in this study can be found in online repositories. The names of the repository/repositories and accession number(s) can be found in the article/**Supplementary Material**.

## Ethics statement

The studies involving humans were approved by Ethics Review Committee of Affiliated Zhongshan Hospital Of Dalian University. The studies were conducted in accordance with the local legislation and institutional requirements. The participants provided their written informed consent to participate in this study.

## Author contributions

Y-CJ: Writing – original draft, Writing – review & editing. Q-TX: Writing – review & editing, Conceptualization, Software, Visualization, Writing – original draft. H-BW: Data curation, Visualization, Writing – review & editing. S-YR: Data curation, Validation, Writing – original draft. YZ: Writing – review & editing, Formal analysis, Supervision.

## Supplementary material

Supplementary Table 1 A curated compilation of 61 published transcriptomic osteosarcoma prognostic models.

Supplementary Table 2 Sequence information of qPCR primers for the five model genes.
